# Supplementary material for: Integral assays of hemostasis in hospitalized patients with COVID-19 on admission and during heparin thromboprophylaxis
Source: PLoS One. 2023 Jun 2;18(6):e0282939. doi: 10.1371/journal.pone.0282939 (PMC10237390; doi:10.1371/journal.pone.0282939)
Supplement: S1 File — (PDF) [file pone.0282939.s001.pdf]

МИНИСТЕРСТВО ЗДРАВООХРАНЕНИЯ РОССИЙСКОЙ ФЕДЕРАЦИИ
Федеральное государственное бюджетное учреждение
НАЦИОНАЛЬНЫЙ МЕДИЦИНСКИЙ ИССЛЕДОВАТЕЛЬСКИЙ ЦЕНТР ДЕТСКОЙ ГЕМАТОЛОГИИ, ОНКОЛОГИИ И ИММУНОЛОГИИ им. ДМИТРИЯ РОГАЧЕВА
(ФГБУ «НМИЦ ДГОИ им. Дмитрия Рогачева» Минздрава России)
117997, Москва, ГСП-7, ул. Саморы Машела, д. 1
тел.: (495) 287-65-70, факс: (495) 664-70-90, E-mail: info@fnkc.ru, www.fnkc.ru
ОКПО 13157861, ОГРН 1027739507212, ИНН/КПП 7728008953/772801001

19.05.2020 № 3э/1-20

На № ____________ от _______________

Выписка из протокола заседания Независимого этического комитета № 3/2020

Дата проведения заседания НЭК: 19.05.2020.

Место проведения заседания НЭК: в режиме Online.

Присутствовали:

Председатель НЭК Володин Н.Н., ответственный секретарь Тарасова И.С., члены НЭК: Вострикова О.В., Жиганова Т.В., Карелин А.Ф., Масчан М.А., Мякова Н.В., Самочатова Е.В., Чернов В.М.

Кворум: есть.

Слушали:

Рассмотрение документов НИР по теме: **«Использование теста Тромбодинамики при COVID-19: определение ранних предикторов развития тяжелой пневмонии и выработка эффективных мер по ее предотвращению»**.

Главные исследователи: академик РАН, д.м.н., проф. Румянцев А.Г., член-корр. РАН, д.б.н., проф. Атауллаханова Ф.И.

Прилагаемые документы:

1. Научная биография главного исследователя члена-корр. РАН, д.б.н., проф. Атауллаханова Ф.И.
2. Протокол НИР: «Использование теста Тромбодинамики при COVID-19: определение ранних предикторов развития тяжелой пневмонии и выработка эффективных мер по ее предотвращению».
3. Информация для пациента и форма письменного информированного согласия пациента
4. Индивидуальная регистрационная карта пациента.
5. Заключение экспертной комиссии от 07.05.2020.

Принятые решения:

Одобрить документы НИР по теме: **«Использование теста Тромбодинамики при COVID-19: определение ранних предикторов развития тяжелой пневмонии и выработка эффективных мер по ее предотвращению»**.

Главный исследователь: академик РАН, д.м.н., проф. Румянцев А.Г., член-корр. РАН, д.б.н., проф. Атауллаханова Ф.И.

Прилагаемые документы:

1. Научная биография главного исследователя члена-корр. РАН, д.б.н., проф. Атауллаханова Ф.И.
2. Протокол НИР: «Использование теста Тромбодинамики при COVID-19: определение ранних предикторов развития тяжелой пневмонии и выработка эффективных мер по ее предотвращению».
3. Информация для пациента и форма письменного информированного согласия пациента
4. Индивидуальная регистрационная карта пациента.
5. Заключение экспертной комиссии от 07.05.2020.

Результаты голосования: «за» – 9 членов, «против» − нет, «воздержавшиеся» – нет.

Председатель НЭК, академик РАН,
профессор, д.м.н. Володин Н.Н.

Рекомендуемые изменения и/или дополнения к документам по теме НИР: **«Использование теста Тромбодинамики при COVID-19: определение ранних предикторов развития тяжелой пневмонии и выработка эффективных мер по ее предотвращению»:**

1. Изменить название темы НИР, поскольку Тромбодинамика не может быть дополнительным критерием диагностики, а позволяет лишь выявить тромботические осложнения у пациентов с COVID-19, убрать из названия «*выработку эффективных мер предотвращения пневмонии*», поскольку исследование носит научный характер и не подразумевает прием препаратов.
2. Предложить в качестве кандидатуры главного исследователя академика РАН, доктора медицинских наук, профессора Румянцева А.Г.
3. Внести исменения в форму информированного согласия (заменить медицинские термины на более доступные для понимания пациентом). Предусмотреть форму информированного согласия для пациентов, находящихся на ИВЛ.
4. Продумать организационные моменты проведения исследования.
5. Получить одобрение ЛЭКов тех медицинских учреждений, в которых будет непосредственно проводиться набор пациентов.
6. Доработать аннотацию темы НИР.
