# Supplementary material for: Integral assays of hemostasis in hospitalized patients with COVID-19 on admission and during heparin thromboprophylaxis
Source: PLoS One. 2023 Jun 2;18(6):e0282939. doi: 10.1371/journal.pone.0282939 (PMC10237390; doi:10.1371/journal.pone.0282939)
Supplement: S2 File — (DOCX) [file pone.0282939.s002.docx]

| 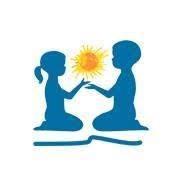 | Ministry of Health of the Russian Federation |
| --- | --- |
|  | The Federal State Budgetary Institution |
|  | Dmitry Rogachev National Medical Research Center Of Pediatric Hematology Oncology and Immunology |
|  | (FSBI NMRC PHOI named after Dmitry Rogachev of the Russian Health Ministry) |
|  | 1 Samory Mashela str., 117997, Moscow, Russia |
|  | Tel.: +7 495 287 65 70, Fax: +7 495 664 70 90,  E-mail: [info@fnkc.ru](mailto:info@fnkc.ru), [www.fnkc.ru](http://www.fnkc.ru) |
|  | OKPO 13157861, PSRN 1027739507212,  ITN/IEC 7728008953/772801001 |

19.05.2020 № 3э/1-20

**Extract from the minutes of the meeting of the Independent Ethics Committee (IEC) № 3/2020**

**Date:** 19.05.2020

**Place:** online

**Present:** Chairman of the IEC Volodin N. N., executive secretary Tarasova I.S., members of IEC: Vostrikova O.V., Zhiganova T. V., Karelin A. F., Maschan M. A., Myakova N. V., Samochatova E. V., Chernov V. M.

**Quorum:** Yes

**Agenda Item:**

Consideration of research documents on the topic: **«Use of thrombodynamics test in COVID-19: identification of early predictors of severe pneumonia and development of effective measures to prevent it»**.

Main researchers: Academician of the RAS, D.M.Sc, professor Rumyantsev A. G. and Ataullakhanov F.I., DSc, Professor, Corresponding Member of the RAS.

Attached documents:

1. Academic biography of main researcher, Corresponding Member of the RAS, DSc, Professor Ataullakhanov F.I.
2. Protocol of research work: «Use of thrombodynamics test in COVID-19: identification of early predictors of severe pneumonia and development of effective measures to prevent it».
3. Information for the patient and patient informed consent form.
4. Individual patient registration card.
5. Conclusion of the expert commission from 07.05.2020.

**Decision:**

Approve documents of research work: **«Use of thrombodynamics test in COVID-19: identification of early predictors of severe pneumonia and development of effective measures to prevent it».**

Main researchers: Academician of the RAS, D.M.Sc, professor Rumyantsev A. G. and Ataullakhanov F.I., DSc, Professor, Corresponding Member of the RAS.

Attached documents:

1. Academic biography of main researcher, Corresponding Member of the RAS, DSc, Professor Ataullakhanov F.I.
2. Protocol of research work: «Use of thrombodynamics test in COVID-19: identification of early predictors of severe pneumonia and development of effective measures to prevent it».
3. Information for the patient and patient informed consent form.
4. Individual patient registration card.
5. Conclusion of the expert commission from 07.05.2020.

**Voting results:** «for» - 9, «against» - no votes, «abstained» - no votes.

Chairman of the IEC, Academician of the RAS,

Professor, D.M.Sc Volodin N. N
