# Supplementary material for: Integral assays of hemostasis in hospitalized patients with COVID-19 on admission and during heparin thromboprophylaxis
Source: PLoS One. 2023 Jun 2;18(6):e0282939. doi: 10.1371/journal.pone.0282939 (PMC10237390; doi:10.1371/journal.pone.0282939)
Supplement: S3 File — (DOCX) [file pone.0282939.s003.docx]

**Application 1**

**Research candidate ID number:_____________**

**Information for the patient**

**The research title:** Individual selection of anticoagulant therapy in patients with a high risk of developing thrombohemorrhagic complications, including those with severe COVID-19 viral infection, ARDS, and severe pneumonia.

This document provides sufficient information about the ongoing scientific research to allow you to decide whether you wish to participate in it. Please read this information carefully and make your decision.

You can discuss the details of this study with your doctor and ask any questions you may have. You can also discuss this study with your family, friends, or other doctors. You will be given time to think. If you choose to participate in this study, you will be asked to sign an informed consent form in duplicate. One signed and dated copy of this information document with the informed consent form will be handed to you, the other will remain with the research physician. If you feel that you are unable to sign the informed consent form, but wish to participate in the study, you may confine yourself to verbal consent. Do not sign this form if you are not satisfied with the answers to your questions and are not sure that you want to take part in this study.

**What is the purpose of the research?**

Changes in hemostasis during COVID-19 and severe pneumonia are associated with an increased risk of developing thrombotic complications, leading to worsening of pneumonia, as well as the development of life-threatening conditions such as sepsis, disseminated intravascular coagulation, thrombosis, and bleeding of various localizations. To prevent these complications or to successfully treat them, reliable laboratory methods for diagnosing hemostasis disorders are needed.The aim of this study is to determine the effectiveness of the use of diagnostic tests of the blood coagulation system to control anticoagulant therapy in patients with a high risk of developing thrombo-hemorrhagic complications, including those with severe COVID-19 viral infection and severe pneumonia.

**Why was I chosen to participate in the study?**

You have been asked to participate in this study because you meet the eligibility criteria for this study.

**Should I agree?**

Participation in scientific research is completely voluntary. If you choose not to participate, it will not affect your continued health care in any way.

**What will happen to me if I give my consent to participate?**

**Stage 1:** You will be asked to complete an informed consent form. If you feel that you are physically unable to sign the informed consent form, you can confine yourself to verbal consent.

**Stage 2:** When prescribing standard blood tests in the clinic for this study, an additional 4.5 ml of venous blood will be taken from you with a thin needle in case you are prescribed a dosage adjustment based on laboratory parameters. Blood sampling will be performed several times, the number of samplings will depend on the duration of your stay in the hospital. Blood sampling will be carried out by qualified medical personnel. Further, this blood will be used to perform a study of the blood coagulation system (hemostasis). The rest of the blood will be disposed of.

**Stage 3:** We will analyze the received data. The data obtained will improve the methods of treatment and prevention of thrombo-hemorrhagic complications.

You will not be prescribed any other medicines, except those necessary for the treatment of your disease and prescribed by your doctor. When you are prescribed anticoagulant therapy, the method of individual selection of the dose of the drug (according to clinical and / or laboratory data) will be determined by randomization. Your attending physician will be informed about the results of your tests/analysis.

**What will happen to me if I do not give my consent to participate?**

Participation in scientific research is completely voluntary. If you choose not to participate, it will not affect your continued health care in any way.

**What will I need to do?**

You do not need to take a direct active part in the Study. If you agree, additional venous blood sampling will be performed on the days of your stay in the hospital on the dates when it is necessary to control and adjust the dose of the drugs as part of your treatment.

**How long will the study take?**

The estimated duration of the study for a particular patient is from 1 week to 1 month, depending on the length of stay in the hospital.

**How many people will take part in the study?**

The estimated number of participants in the study is 500 people.

**Are you exposed to any risks by taking part in this study?**

Enoxaparin sodium and nadroparin calcium are anticoagulant drugs that have a number of side effects. Therapy with these drugs will be prescribed to you as part of your treatment, regardless of your participation in this study. The results of laboratory tests will influence the process of selecting an individual dose of these drugs. Your doctor will carefully review your medical history and laboratory test results to minimize the risk of side effects from these drugs.

Also, there are risks of additional blood sampling. As with any blood sampling, there is the possibility of mild pain, bruising, or infection at the puncture site. All necessary measures will be taken to avoid this.

Only disposable sterile blood collection systems will be used during the study.

Your participation in the study, all data and results of the study are protected by medical secrecy and are treated strictly confidentially.

**Will my data or tissue samples from my body be used or stored for future purposes?**

Your plasma sample can be stored in a special archive for at least 10 years. During this time, additional research may be carried out as a result of new scientific developments. The information obtained in the course of this study will be available to the members of the study group, the ethics committee and regulatory organizations. By signing this document, you agree that anonymized data from this study may be published in scientific sources, transferred to other research groups without obtaining additional consent from you. We guarantee the confidentiality of personal information and respect for your civil rights.

**Do I have to consent now to the possible future use of my data or tissue samples from my body (do I need separate information or separate consent for this)?**

You will not be required to sign any additional consents.

**Can I withdraw my consent during the study?**

Yes, the study is completely voluntary at all times.

**What happens if I withdraw my consent?**

If you decide not to participate and revoke previously signed or verbally approved consent, this will not affect your future health care in any way. Plasma samples will not be kept for further research.

**What therapy/procedure/etc. will be studied?**

The possibilities of laboratory tests of the blood coagulation system (including the D-dimer test, thrombodynamics, thromboelastography, etc.) for individual selection of anticoagulant therapy will be explored. These tests are laboratory tests and do not require any clinical procedures other than blood sampling.

**What are alternative methods of diagnosis/treatment?**

In the study, along with hemostasis tests, clinical and instrumental data will be used, which will also be used by your doctor to assess your condition and correct therapy. An alternative to dosing based on laboratory tests is standard therapy with fixed doses of anticoagulants calculated by body weight.

**Will I incur any costs associated with participating in the study?**

All additional diagnostic tests related to the study, including laboratory tests, will be provided free of charge. No payments are provided. This study is of exclusively scientific interest, commercial use of the obtained data is not provided.

**Will I be informed of any randomly discovered data?**

In case of detection of significant changes in the coagulation system according to standard tests, your attending physician will correct the violations in accordance with the official clinical and methodological recommendations.

**What are the possible benefits of participating?**

If the applicability of the obtained data is found, the existing recommendations for the administration of anticoagulant therapy in patients with COVID-19 and severe pneumonia will be corrected.

**What if new information emerges during the course of the study?**

Any new information will be additionally reported during the course of the study.

**What happens when the study ends?**

Participation in this study does not imply the need for the patient to stay in the hospital for additional time, the length of the patient's stay in the hospital does not depend on the results or duration of the study. At the end of the study, all clinical and laboratory data will be analyzed and, based on them, printed articles will be written in scientific journals.

**Will I continue to receive medical care?**

Yes. Participation in this research study will not affect your current or future medical care in any way.

**What will be done in the event of an emergency?**

It is highly unlikely that an emergency situation arising in connection with the study will occur. If such a situation does arise, the necessary measures will be taken to eliminate it. Since the study is voluntary at all times, you can withdraw your consent to participate in the study at any time.

**What will happen to the research results?**

Based on the data received, abstracts of conferences and printed articles will be published.

**Will I be informed about the results?**

The results of the study will be available after they are published.

**Who organizes and finances the research?**

The study was organized by the City Clinical Hospital No. 40 DZM in collaboration with Dmitry Rogachev National Medical Research Center Of Pediatric Hematology, Oncology and Immunology with the support of HemaCore LLC (provision of instruments and reagents for the thrombodynamic test). Supervision of the study is carried out by the staff of the Dmitry Rogachev National Medical Research Center Of Pediatric Hematology, Oncology and Immunology.

**Will I be paid to participate in the study?**

There are no payments during the course of the study.

**For more information, please contact:**

**INFORMED CONSENT FORM**

The study and the consent form were explained to me. I have read all pages of the study participant information and have had the opportunity to ask questions to help me understand what my participation will be. All my questions have been answered satisfactorily.

I voluntarily agree to participate in the scientific study «Individual selection of anticoagulant therapy in patients with a high risk of developing thrombohemorrhagic complications, including those with severe COVID-19 viral infection, ARDS, severe pneumonia».

I am warned that, according to the results of laboratory tests, the doses of drugs administered to me (low molecular weight and unfractionated heparins) may differ from those indicated in the instructions for the drugs.

I have received a signed and dated copy of this informed consent form.

I authorize the use of information from medical records for scientific purposes in the framework of this study with confidentiality (in an anonymized form).

I agree that my health information, including laboratory test results, may be used for future medical research in an anonymous manner.

I understand that I can voluntarily withdraw from the study at any time and this will not affect my continued medical care.

By signing this consent form, I do not waive any statutory rights that I might have as a research subject.

__________________________________________

Full name

__________________________________________ _______________

Signature Date
